# Development and validation of an open source Monte Carlo dosimetry model for wide‐beam CT scanners using Fluka

**DOI:** 10.1002/acm2.12559

**Published:** 2019-03-09

**Authors:** Elanchezhian Somasundaram, Nathan S. Artz, Samuel L. Brady

**Affiliations:** ^1^ Department of Diagnostic Imaging St Jude Children's Research Hospital Memphis TN USA; ^2^ Department of Radiology Cincinnati Children's Hospital Medical Center Cincinnati OH USA; ^3^ Department of Radiology University of Cincinnati Cincinnati OH USA

**Keywords:** CT, Fluka, Monte Carlo simulation, organ dosimetry, radiation dose

## Abstract

**Purpose:**

Development and validation of an open source Fluka‐based Monte Carlo source model for diagnostic patient dose calculations.

**Methods:**

A framework to simulate a computed tomography (CT) scanner using Fluka Monte Carlo particle transport code was developed. The General Electric (GE) Revolution scanner with the large body filter and 120 kV tube potential was characterized using measurements. The model was validated on benchmark CT test problems and on dose measurements in computed tomography dose index (CTDI) and anthropomorphic phantoms. Axial and helical operation modes with provision for tube current modulation (TCM) were implemented. The particle simulations in Fluka were accelerated by executing them on a high‐performance computing cluster.

**Results:**

The simulation results agreed to better than an average of 4% of the reference simulation results from the AAPM Report 195 test scenarios, namely: better than 2% for both test problems in case 4 using the PMMA phantom, and better than 5% of the reference result for 14 of 17 organs in case 5, and within 10% for the three remaining organs. The Fluka simulation results agreed to better than 2% of the air kerma measured in‐air at isocenter of the GE Revolution scanner. The simulated air kerma in the center of the CTDI phantom overestimated the measurement by 7.5% and a correction factor was introduced to account for this. The simulated mean absorbed doses for a chest scan of the pediatric anthropomorphic phantom was completed in ~9 min and agreed to within the 95% CI for bone, soft tissue, and lung measurements made using MOSFET detectors for fixed current axial and helical scans as well as helical scan with TCM.

**Conclusion:**

A Fluka‐based Monte Carlo simulation model of axial and helical acquisition techniques using a wide‐beam collimation CT scanner demonstrated good agreement between measured and simulated results for both fixed current and TCM in complex and simple geometries. Code and dataset will be made available at https://github.com/chezhia/FLUKA_CT.

## 1 INTRODUCTION

The use of radiation based imaging systems has become indispensable for medical diagnosis leading to a rise in diagnostic radiation. However, long term health effects of cumulative diagnostic radiation exposure are not yet completely understood. The evaluation of possible long‐term effects from diagnostic radiation will only be possible once fast and accurate patient‐specific dose quantification is available. Current practice simply archives machine‐dependent, radiation output reports containing examination‐specific and phantom‐based dosimetry information such as volume computed tomography dose index (CTDI_vol_) and dose length product (DLP). Calculations exist to convert these phantom‐based radiation output metrics to dose estimates more representative of patient dose, such as size specific dose estimates (SSDE)^1^, ^2^ which have been used to estimate organ specific doses^3^, ^4^ and population‐specific effective dose from SSDE and DLP.^5^ These techniques, however, are still based on approximations and are not patient‐specific. Since patient size, anatomy, geometry, and tissue composition can vary greatly among patient populations, even within similar ages and weight groups, quantifying patient‐specific radiation dose is highly preferable.

For radiation transport problems with complex geometry and varying material properties, Monte Carlo simulation‐based dose quantification techniques remain the gold standard despite long computational times. With the recent advent of lower‐cost high performance computing resources, using Monte Carlo simulations for patient‐specific radiation dose reporting within the clinical environment is more promising than ever before. Additionally, recent studies have implemented Monte Carlo based dose simulations in parallel computing environments, such as Graphical Processing Units (GPUs).^6^, ^7^ The implementation of high‐performance, parallel computing may lead to patient‐specific radiation dosimetry calculation times that become feasible even during demanding clinical schedules.

The purpose of this study is to develop a fast, multi‐mode (i.e., helical and axial) CT scanner simulation model of a wide‐beam CT scanner including tube current modulation (TCM). The radiation transport model for the CT scanner is developed using the open source Monte Carlo code Fluka. Although previous studies have utilized Fluka in applications such as radiotherapy^8^ and nuclear medicine,^9^ Fluka has not been validated for diagnostic dose calculations in CT. As the same voxel geometry and organ segmentation generated for radiotherapy dose simulations can be used for diagnostic dose calculations, the model developed in this work can be extended to calculate total radiation dose for radiotherapy patients using Fluka.

The CT scanner model developed in this work is validated on computational benchmark problems and experimental test problems that measure in‐air exposure at the isocenter, air kerma in the CTDI phantom, and direct organ dose measurements in a physical anthropomorphic phantom for the General Electric (GE) Revolution CT scanner. Additionally, the CT source model is designed to take scanner‐specific input parameters and will be made available as open‐source code along with all the preprocessing and postprocessing routines necessary to model any CT scanner in Fluka and execute them in a high performance cluster.

## 2 MATERIALS AND METHODS

### 2.A CT source modeling

#### 2.A.1 Fluka Monte Carlo particle transport code

Fluka is a general purpose particle transport calculation tool that can track a wide range of charged and neutral particles including photons and electrons with energies ranging from 100 eV to thousands of TeV. Fluka also includes stable physical models to handle various particle interaction mechanisms across a variety of material types. Fluka (Version 2011.2c‐5) supports complex geometries through a combinatorial geometry package and has options for user written routines that were used to customize the simulation to suit specific problems in this study. The source routine in Fluka was highly modified to support various operation modes of the CT scanner, for example, axial and helical. The input simulation files for benchmarking Fluka accuracy were generated using an advanced user interface called Flair. Flair has an inbuilt routine that allows voxelization of CT images into a format that is understood by Fluka.

#### 2.A.2 Source spectrum generation

The energy spectrum of the x‐ray tube for the GE Revolution CT scanner was not readily available from the vendor as it was proprietary information. To generate the source x‐ray photons for the Monte Carlo simulation, the tube x‐ray spectrum was calculated using SPEKTR 3.0^10^ and optimized to match the measured spectrum of the GE Revolution scanner. The SPEKTR toolkit calculated x‐ray spectra based on the Tungsten Anode Spectral Model using Interpolating Cubic Splines (TASMICS) algorithm. It generated the x‐ray spectra in 1 keV energy bins over beam energies from 20 to 150 keV. The spectrum calculated from SPEKTR was given in photons/mm^2^/mAs at 100 cm from the source. The spectrum at the isocenter of the scanner (62.56 cm from the tube for the GE Revolution) was then calculated by accounting for geometric attenuation. The TASMICS spectrum from SPEKTR represented the unfiltered spectrum from the x‐ray tube. In modern CT scanners, the x‐ray spectrum undergoes some inherent filtration after exiting the x‐ray tube housing and to determine this filtered spectrum, SPEKTR included an optimization function that matched the exposure of the final spectrum to the measured x‐ray tube exposure (mGy/mAs). In this study, the SPEKTR generated spectrum was optimized to match both exposure and Half Value Layer (HVL) of the measured spectrum using MATLAB's [2016a] optimization toolbox. The optimization involved generating an equivalent filter that provides a filtered spectrum that matches the measured spectrum.^11^ The x‐ray spectrum at 120 kV tube potential measured at the iso‐center of the scanner was optimized with an equivalent filter made of carbon and aluminum with 3.6 and 9.3 mm thickness, respectively. The electron and photon cut‐off energies for the Fluka simulation were set to be 1 and 0.5 KeV for all material regions.

#### 2.A.3 Bowtie filter characterization

In addition to the inherent tube filtration, a bowtie shaped filter is used to absorb the lower energy photons of the spectrum such that the periphery of the body is not over exposed to radiation and to maintain uniform image quality across the scan volume. The shape of the bowtie filter was characterized by measuring the beam intensity along the radial axis of the scanner gantry.^11^ The tube was parked at 90° using the service mode of the scanner and a 0.6 cc ion chamber (10x6‐0.6, Radcal, Monrovia, CA) was mounted on a jig placed on the patient table such that the center of the chamber aligned with the isocenter of the scanner. Measurements of the in‐air exposure were then made at intervals of 1 cm from isocenter by adjusting the table height upwards until the ion chamber was at the edge of the bowtie filter towards the top of the CT gantry. The measured exposure values were normalized and used to optimize the thickness profile of the bowtie filter such that the equivalent spectrum matched that measured spectrum along the radial axis. Once the thickness profile of the bowtie was determined, the filtered spectrum exiting the bowtie at each of the 1 cm intervals was calculated. Based on the total number of photons emitted in each bowtie interval, a discrete probability distribution function of the bowtie profile was created. During the Monte Carlo simulation in Fluka, the initial direction of the x‐ray photons was sampled using the bowtie distribution function. The bowtie interval, through which the particle would exit the filter, was sampled from the probability distribution function, and uniform random sampling was used within the interval to select the exact coordinates (XY) that would be traversed by the exiting particle. The initial polar angle of the particle was then calculated from the XY coordinates.

#### 2.A.4 Beam profile characterization

The heel effect, that is, the variation in beam intensity (or beam profile) along the long axis (z‐axis) perpendicular to the radial axis of the scanner, was more pronounced with the GE Revolution scanner when operated using wide collimation (up to 16 cm) compared to previous publications covering only 4 cm along the z‐axis.^12^ The beam profile was measured by exposing a radiochromic film at the desired collimation with 375 mA, 120 kV and 1 s tube rotation. Exposure values in the range between 350 to 375 mAs were optimal for beam width measurements using radiochromic film and were verified using ion‐chamber measurements.^13^ The exposed film was digitized using an Epson flatbed scanner (10000XL) and processed in a custom‐built MATLAB [2016a] script to determine the FWHM of the beam profile. The beam profile FWHM was considered the actual collimation width for the simulation, and was discretized into 1 cm intervals and converted into a discrete probability distribution of the x‐ray intensity. The probability function was used to sample the z‐coordinate, and the initial azimuthal angle, with which the source photons would reach the patient volume. Combining the initial azimuthal angle along with the polar angle, sampled from the bowtie profile distribution, and yielded the initial particle direction of the source photons.

#### 2.A.5 Tube rotation and table movement

The actual tube rotation in a scanner can usually be approximated as small fixed steps around the circumference of the gantry, but the exact number of steps to complete a single rotation was unavailable. Therefore, in this study, the source photons were sampled uniformly along the circumference of the rotation path with the tube‐to‐isocenter distance as the radius.

For helical scans, the table movement was simulated by translating the x‐ray tube along the z‐axis with the table and the patient held at a fixed position. This was achieved by uniformly sampling the z‐location for the source particle along the range of table movement with respect to the x‐ray tube. The range of the table movement *T*_*z*_ was given by:

(1) $$T_{Z}=\frac{P*Col*Exp_{\mathrm{time}}}{Rot_{\mathrm{time}}},$$

where, *P* was the pitch of the helical scan, *Col* was the x‐ray tube collimation, *Exp*_*time*_ was the total exposure time for the scan and *Rot*_*time*_ was the time taken by the tube to complete one full rotation. The z‐location for the source particle, *z*_*p*_ was sampled as:

(2) $$z_{p}=z_{i}\pm T_{z}*\epsilon ,$$

where, *ε* was a random number between 0 and 1, *z*_*i*_ was the starting location of the tube with respect to the patient/table. The ± sign was used to indicate that the table movement could be in the positive or negative direction depending on a head first or feet first scan. Once *z*_*p*_ was sampled, the radial location (*x*_*p*_, *y*_*p*_) of the particle was determined as:

(3) $$x_{p}=R_{\mathrm{iso}}*\sin\left(\beta_{p}\right),\;y_{p}=R_{\mathrm{iso}}*\cos\left(\beta_{p}\right),$$

Where, *R*_*iso*_was the tube to isocenter distance for the scanner and *β*_*p*_ was calculated as:

(4) $$\beta_{p}=\beta_{i}+\frac{|z_{p}-z_{i}|*2\pi }{P*Col},$$

*β*_*i*_ was the initial angle (in radians) made by the x‐ray tube with respect to the *y*‐axis of the scanner.

#### 2.A.6 Tube current modulation (TCM)

The parametric details to simulate TCM were not available in the DICOM header metadata. To model TCM in this study, the average beam current (mA) recorded for each reconstructed slice was used to construct a probability distribution along the z‐axis of the scan and the source photons were sampled from this distribution to simulate the TCM profile. The TCM algorithm implemented in the Fluka model did not simulate the rotational beam current modulation within each reconstructed slice (i.e., modulation in the x‐y plane).

### 2.B Primary fluence calculation

The primary x‐ray fluence of the x‐ray tube derived from Fluka was tallied as dose per primary particle and this quantity was scaled by the total number of primary photons emitted based on kV, mA, scan duration, filter settings, collimation, and heel effect. The total number of primary photons was used to estimate the total simulated dose/exposure for comparison with the measured dose/exposure values to validate the simulation model. The following formula was used to calculate the total source strength (Q):

(5) $$Q=\sum_{i=1}^{N_{B}}\sum_{j=1}^{N_{H}}H_{j}\;A_{\mathrm{ij}}{\left(\frac{D_{\mathrm{ij}}}{\mathrm{SDD}}\right)}^{2}\sum_{k=1}^{N_{K}}Q_{i,k,mAs,Kvp,SDD},$$

where, *N*_*B*_ was the total number of bins used to characterize the bowtie filter, *N*_*H*_ was the total number of bins used to characterize the beam profile, *H*_*j*_ was the normalized beam intensity in bin j, *N*_*K*_ was the total number of energy groups handled by SPEKTR, *A*_*ij*_ was the rectangular area mm^2^ formed by the length of the bowtie filter bin *i* and length of the beam profile bin *j*,* D*_*ij*_ was the distance from the x‐ray tube to the center of the rectangular area *A*_*ij*_, *SDD* was the distance from the x‐ray tube to isocenter of the scanner, and finally, *Q*_*i*,*k*,*mAs*,*Kvp*,*SDD*_ was the number of primary photons measured at the isocenter per square millimeter filtered by the bow tie filter thickness in bin *i*, in energy group k, for the *mAs* at the specified *kV*.

### 2.C Material properties and geometry for simulation

**Figure 1:**
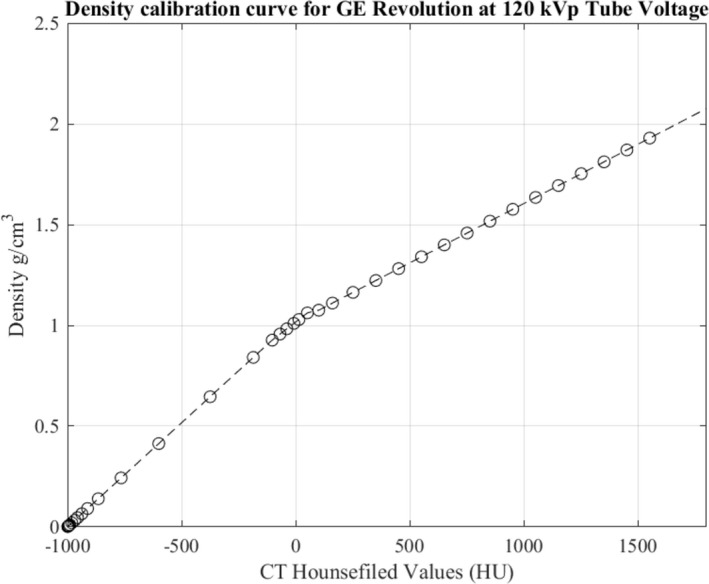
The calibration curve mapping Hounsfield unit values to material density calculated using phantom measurements and interpolation functions described in Ref. 14.

To perform Monte Carlo particle simulations on phantoms, the scanned images in DICOM format were converted into voxel geometry within Flair, and the Hounsfield values of the voxels were converted into corresponding elemental weights and densities^14^ that split the Hounsfield scale into four different tissue groups, namely: air‐fat, fat‐water, soft tissue, and skeletal tissue. Within each group the density and elemental weights were interpolated for selected tissue types. The coefficients for the interpolation functions were derived from measured Hounsfield values for a total of 22 known materials obtained by scanning a Gammex Sensitometry phantom (CT Electron Density phantom, Sun Nuclear Corp., Middleton, WI) and a CatPhan 700 Phantom (Phantom Laboratory, Greenwich, NY). The interpolation functions were used to determine the conversion maps to calculate densities and elemental weights for the entire range of Hounsfield values (HU) in the DICOM images. Figure 1 shows the density calibration curve calculated for the GE revolution with phantom measurements made at a tube voltage of 120 kVp, while Table 1 shows the material composition in each HU range.

**Table 1:** Material composition calibration as function of HU range for General Electric Revolution expressed as weight fraction

HU min	HU max	Hydrogen	Oxygen	Carbon	Nitrogen	Chlorine	Calcium	Phosphorous	Magnesium
−1500	−938	0.000	0.237	0.000	0.765	0.000	0.000	0.000	0.000
−937	−104	0.103	0.749	0.105	0.031	0.003	0.000	0.002	0.002
−103	−70	0.116	0.187	0.693	0.001	0.001	0.000	0.000	0.000
−69	−40	0.113	0.308	0.566	0.009	0.001	0.000	0.000	0.000
−39	−9	0.110	0.409	0.460	0.015	0.002	0.000	0.001	0.000
−8	13	0.108	0.508	0.357	0.022	0.002	0.000	0.001	0.000
14	50	0.106	0.575	0.287	0.026	0.002	0.000	0.001	0.000
51	100	0.103	0.723	0.134	0.030	0.002	0.000	0.002	0.000
101	160	0.094	0.622	0.207	0.062	0.003	0.000	0.000	0.000
161	250	0.094	0.356	0.451	0.025	0.001	0.047	0.022	0.000
251	350	0.088	0.364	0.419	0.027	0.001	0.066	0.031	0.001
351	450	0.081	0.373	0.387	0.029	0.001	0.086	0.040	0.001
451	550	0.075	0.381	0.358	0.030	0.001	0.103	0.048	0.001
551	650	0.070	0.388	0.331	0.032	0.001	0.119	0.055	0.001
651	750	0.065	0.395	0.306	0.033	0.001	0.134	0.062	0.001
751	850	0.060	0.401	0.284	0.035	0.001	0.148	0.068	0.001
851	950	0.056	0.406	0.263	0.036	0.000	0.160	0.074	0.001
951	1050	0.052	0.411	0.244	0.037	0.000	0.172	0.079	0.002
1051	1150	0.048	0.416	0.226	0.038	0.000	0.182	0.084	0.002
1151	1250	0.045	0.420	0.209	0.039	0.000	0.192	0.088	0.002
1251	1350	0.042	0.425	0.194	0.040	0.000	0.202	0.092	0.002
1351	1450	0.039	0.428	0.179	0.041	0.000	0.210	0.096	0.002
1451	1550	0.036	0.432	0.166	0.041	0.000	0.219	0.100	0.002
1551	1800	0.034	0.436	0.153	0.042	0.000	0.226	0.104	0.002
1801	2000	0.028	0.443	0.124	0.044	0.000	0.243	0.111	0.002

The exact geometry of the patient table was proprietary information so it was modeled based on a thin slice, high tube current CT scan of the table. Preprocessing routines were employed to convert the table HU values into corresponding carbon fiber and foam material properties. All CT images were processed to remove the incomplete table reconstruction that occurs due to the table being outside the reconstructed field of view. These images were then superimposed onto the high quality CT scan of the table such that the resulting image contained the patient/phantom volume along with a full representation of the CT table.

### 2.D Test problems

#### 2.D.1 AAPM Report 195 benchmark tests

To validate Fluka, the source model, and other routines developed for the CT scanner were used to simulate the benchmark problems described in cases 4 and 5 of the AAPM Report 195.^15^ A mono‐energetic 56.4 keV photon source and a tungsten‐aluminum (W/Al) 120 kVp spectral source were used for both problems.

For case 4, Fluka simulations were performed based on the 32 cm diameter CTDI body phantom geometry. For test 1, the x‐ray tube was held static at 0° from isocenter, and for test 2, a rotating tube with random sampling of source photons along the circumference of the gantry was simulated. Both tests for case 4 were run for a large particle history (10^8^) such that the statistical errors were to better than 1%. The simulation geometry for case 4 test 1 and test 2 are shown in figs. 14–15 (pages 25–26) and fig. 16 (page 27), respectively of AAPM Report 195 and the corresponding simulation parameters for tests 1 and 2 are given in Table 2.

**Table 2:** Simulation parameters for the TG195 Case 4 test problem used in this work

Parameter	Values	Parameter	Values
Collimation	10 mm, 80 mm	Scan field of view	320 mm
Photon source	Mono 56.4 keV, spectral 120 KVp (W/Al)	Tube to isocenter distance	600 mm
Tube position — test 1	Fixed — 0°	Tube Position — test 2	Random rotation
Scoring — test 1	4 contiguous cylinders (fig. 15; report 195)	Scoring — test 2	Two 10 mm cylinders (fig. 16 report 195)

For case 5, the geometry of the source model was the same as case 4 except the PMMA phantom was replaced by a voxelized phantom with 17 different organs that were used to tally the energy deposition from the scan. The voxelized phantom had dimensions of 320 × 500 × 260 voxels where each voxel was 1.0 mm × 1.0 mm × 1.0 mm in size. Two test problems were simulated, one with a static tube at 0° and the second with a rotating tube, similar to case 4. The simulation parameters are shown in Table 3, and the geometry setup in fig. 17 of AAPM Report 195 (pages 28). Fluka simulations in the report for the static acquisition at 0° were performed for 10^8^ photons. The statistical errors remained within 2% for 16 of the 17 organs in the XCAT phantom for both monoenergetic and spectral sources. The adrenal gland had errors of 4.2% and 5.5%, respectively for the monoenergetic and spectral sources. For the random rotating tube acquisition, the statistical errors for a particle history of 10^8^ remained within 1% for all the organs except the adrenal gland (~4%) and thyroid (~1.5%) for both sources. Simulation results were compared with four other production codes provided in AAPM Report 195, namely: MCNP5, Penelope, Geant4 and EGSnrc for static tube configurations, and for rotating tube configurations, Fluka results were compared to the results from Geant4 and MCNP5.

**Table 3:** Simulation parameters for the TG195 Case 4 test problem used in this work

Parameter	Values	Parameter	Values
Collimation	10 mm	Scan field of view	500 mm
Photon source	Mono 56.4 Kev, Spectral 120 KVp (W/Al)	Tube to isocenter distance	600 mm
Tube Position — test 1	Fixed — 0°	Tube position — test 2	Random rotation
Scoring — test 1 and 2	17 organs (voxel regions)		

#### 2.D.2 In‐air exposure measurements and simulation

To validate the Fluka source model for the GE Revolution CT scanner, in‐air exposure measurements made at the isocenter of the scanner using a Radcal 3 cc pencil chamber (10x6‐3CT, Radcal, Monrovia, CA) and were compared to the simulation results at 80 and 160 mm beam collimations. The scanner was operated in a fixed, nonrotating gantry geometry within service mode, and the measurements were made for fixed tube current, voltage, filter, and focal spot settings as shown in Table 4. The mean of three repeated measurements was considered as the measured exposure/dose for all test problems in this work.

**Table 4:** Scanner settings for in‐air exposure measurements and computed tomography dose index phantom measurements

Parameter	Values	Parameter	Values
Collimation	80 mm, 160 mm	Scan field of view	500 mm
Tube voltage	120 KVp	Tube to isocenter distance	625.6 mm
Tube current	300 mA	Filter	Large body
Tube rotation	1 s	Focal spot	Small

**Figure 2:**
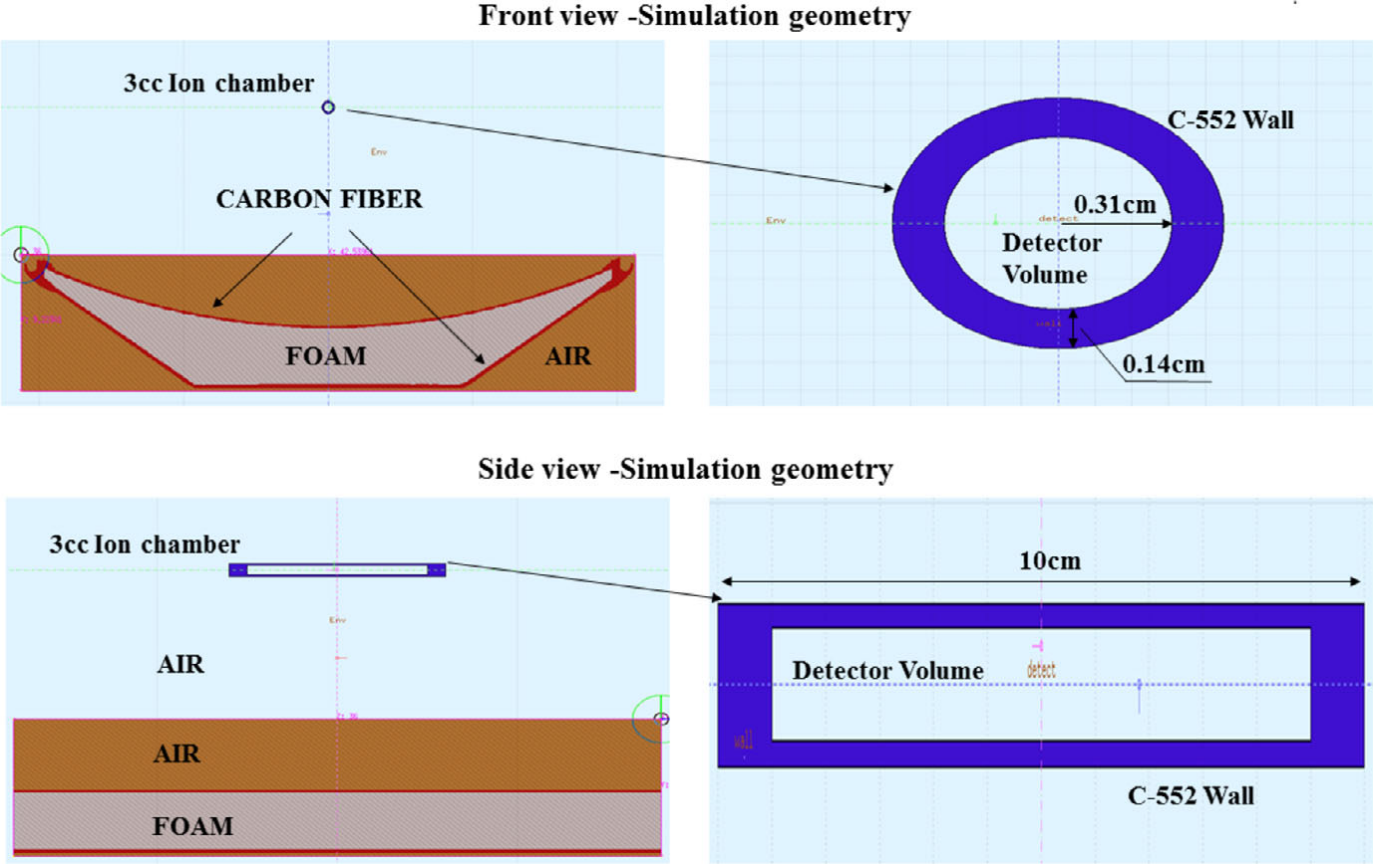
*Simulation setup for 3 cc ion chamber Air Kerma measurement at isocenter*.

Figure 2 shows the measurement setup and the simulation setup in Fluka for this study. For the simulation, the pencil chamber was modeled as a simple cylinder without accounting for stem and other related electronics. The patient table was included in the geometry by placing a voxelized volume generated from a scan of the table using Flair. The air volume within the pencil chamber was assigned as the detector tally, and the energy fluence of the photons was tallied in 30 energy bins. The energy fluence values were converted to exposure values using the mass energy attenuation factors generated from NIST XCOM photon cross section databases.^16^ The method of tallying the energy fluence instead of air kerma (exposure) directly helped speed‐up the simulation by allowing the use of a smaller particle history size of 10^7^ compared to ~10^9^ while maintaining the statistical precision to better than 1% for all the test cases.

#### 2.D.3 CTDI phantom measurements and simulation

**Figure 3:**
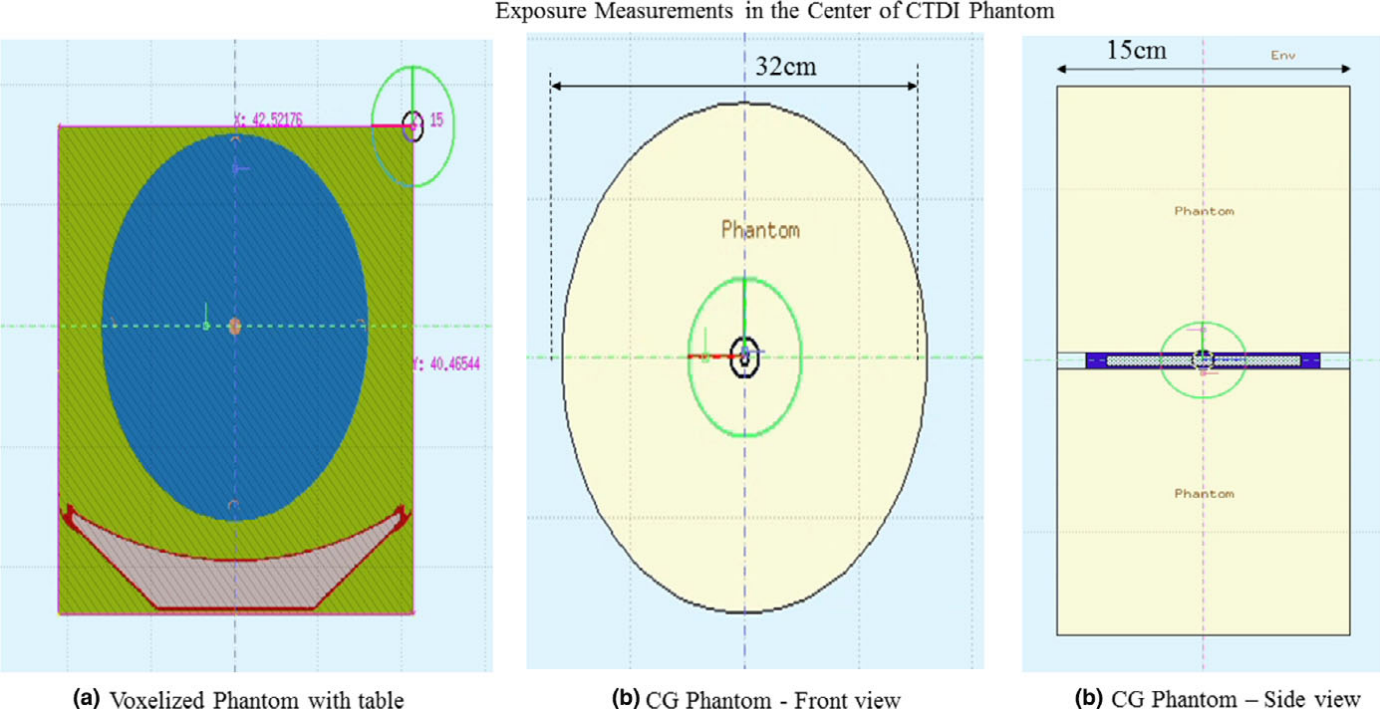
(a) Computed tomography dose index phantom simulation model using voxelized geometry. (b) CTDI phantom and pencil chamber in combinatorial geometry (CG).

To test the performance of the proposed Fluka source model in simulating a CTDI measurement experiment, a 15 cm long CTDI body phantom (32 cm diameter) was exposed using the same scanner settings in Table 4. The exposure measurements were made using the pencil chamber at the center hole of the phantom. The CTDI phantom and the pencil chamber geometry were modeled using combinatorial geometry in Fluka. The voxelized table used for the in‐air exposure simulations could not be positioned below the CTDI phantom as the cylindrical PMMA region of the phantom overlapped with the air region in the voxelized volume. Therefore, to account for the effect of table attenuation, two simulations were performed: (a) A scan of the CTDI phantom without the ion chamber was voxelized such that the solid part of the phantom was assigned to PMMA material while the center hole in the phantom was assigned to air, which was also used as the detector tally for the Monte Carlo simulation; (b) The same simulation was repeated on a voxelized volume containing both the table and the phantom by superimposing the table scan and the phantom scan [Fig. 3(a)]. The difference in the detector tally with and without the table was used to correct the simulated exposure in the pencil chamber modeled using the combinatorial geometry [Fig. 3(b)]. The measured exposure values were then compared to the simulated exposure with the correction for table attenuation. The simulations were performed for a particle history of 10^7^ with a precision less than 1%.

#### 2.D.4 Physical anthropomorphic phantom measurements and simulation

**Figure 4:**
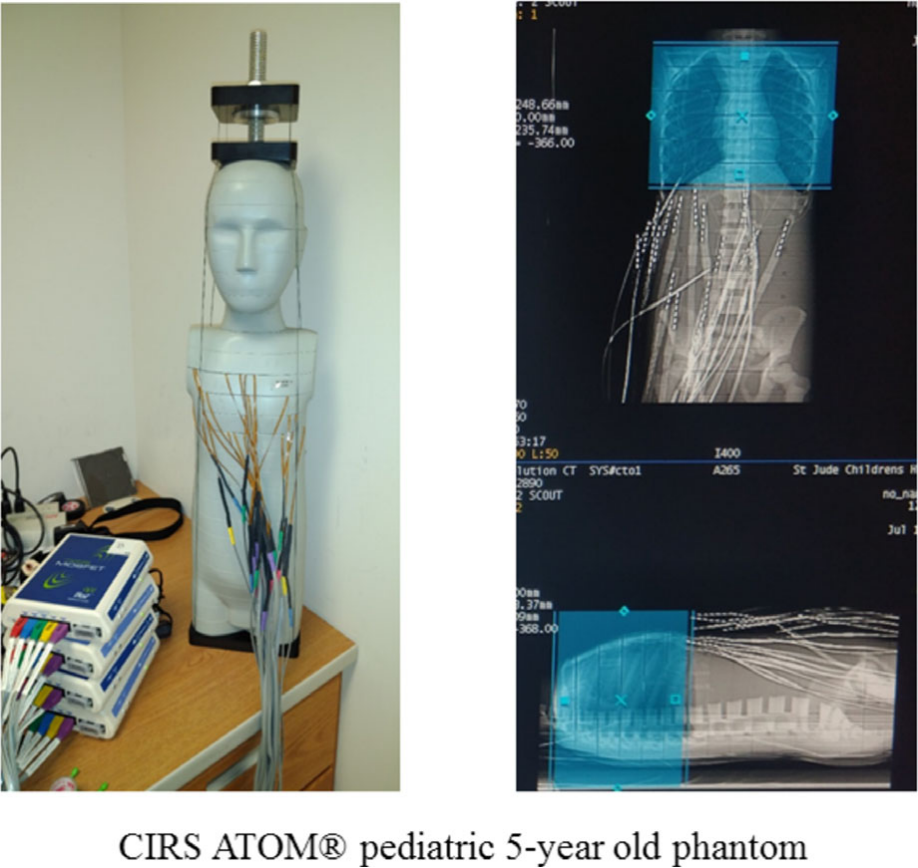
CIRS pediatric 5‐yr‐old phantom (left) along with the corresponding scan range (shown as blue box superimposed over the planar radiograph of the phantom) used for dosimetry with MOSFET detectors.

To test the accuracy of this Fluka simulation model that incorporates voxelized volumes derived from scanned images, organ radiation dose values were simulated and compared with high‐bias MOSFET detectors (MobileMOSFET, BEST Medical Canada, Ottawa ON) within a pediatric 5‐yr old anthropomorphic phantom (ATOM, CIRS, Norfolk VA) for both axial and helical acquisition modes. Figure 4 demonstrates the phantom fully loaded with MOSFET dosimeters, and the respective prescribed CT scan range.

The 25 MOSFETs were placed within lung, soft tissue, and bone organ structures spanning the thorax region. For axial mode, the scan was performed with a single 160 mm beam collimation. For helical mode, scans were performed with a beam collimation of 80 mm using both TCM as well as fixed current modes. CT scan acquisition parameters for both modes were shown in Table 5; for the helical scan with TCM, the mA values varied between 150 and 450 mA and the Noise Index of the image was 4.

**Table 5:** Scan settings used for the pediatric 5‐yr‐old phantom for axial and helical modes

Parameter	Values	Parameter	Values
Tube voltage	120 kV	Scan field of view	500 mm
Tube current	300 mA	Tube to isocenter distance	625.6 mm
Focal spot	Small	Filter	Large body
Pitch (helical)	1.531	Pitch (axial)	1 s
Exposure time (helical)	1.05 s	Exposure time (axial)	1 s
Collimation (helical)	80 mm	Collimation (axial)	160 mm
Tube rotation (helical)	0.5 s	Tube rotation (axial)	1 s

The MOSFET detectors were cross‐calibrated with an x‐ray machine using the methods previously described.^17^ The HVL of the x‐ray machine was matched to that of the CT machine used by placing additional aluminum plates at the exit port of the x‐ray machine to harden the x‐ray beam for a HVL of 7.62 mm Al at 120 kV. The average calibration error was 10% (range 4%–16%) for the 25 MOSFET detectors. The measured output from the MOSFET detectors were multiplied by the MOSFET calibration correction factors and tissue equivalent conversion factors for air and lung, bone, and soft tissue^18^ to yield the actual dose deposition to the tissues.

For the Fluka simulation, the phantom without the MOSFET detectors was scanned and the images were used to generate the voxelized volume. The images from the scan with the loaded MOSFET detectors were registered to the voxelized phantom and the MOSFET location coordinates were mapped to the voxelized phantom coordinate system to determine the tally locations within the simulation geometry. Cylindrical tally regions of radius 2 mm and length 10 mm were used to estimate the dose deposition at the location of the MOSFET detectors. The active region of the MOSFET detectors was small (0.2 mm × 0.2 mm × 0.2 mm) in comparison to the tally used. The simulations were run for 10^8^ photons and the tally size was chosen such that they were large enough to achieve a statistical precision to better than 7%, and small enough such that they did not overlap with other neighboring tissues; since the MOSFET measurement errors had a standard deviation of ~10%, a more accurate simulation was not required.

The MOSFET measurements were made for three consecutive scans and the results were averaged. The standard deviation of the measurement dose was calculated by propagating the MOSFET calibration errors. For simulated doses in Fluka, the statistical errors in the simulation were used as an estimate for standard deviation. The tube starting position in the XY coordinates was not available from the DICOM header, and although this did not affect the axial scans, it would have had an effect on the dose values for helical scans. To negate this effect, four simulations with different tube starting positions (0°, 90°, 180°, and 270° to the isocenter) were performed and the dose values were averaged for helical scans. The standard deviation for the averaged dose was calculated by propagating the statistical errors in each simulation.

### 2.E Parallel execution of Fluka

The high performance computing facility (HPCF) at St. Jude Children's Research Hospital had close to 5000 CPU cores and 41 TB of Random Access Memory (RAM) with IBM SONAS as the primary storage environment supporting up to 3 PB of storage capacity. All the computer nodes were connected through a 40 Gb/s interconnect. The HPCF resources were managed through the load sharing facility (LSF) software from IBM. To execute Fluka in parallel, a bash script was developed. The bash script, when launched, modified the base input deck for the simulation by assigning randomly generated random number seeds and submitted the job for execution in parallel to as many cores as requested by the user with the necessary memory allotment for the job. Once the jobs were executed, a postprocessing script piped the output files generated by each CPU core through Fluka's postprocessing utility. In this study, a total of 100 cores were used to launch all simulation problems, using more cores would sometimes result in queuing depending on the workload on the HPCF. The scaling in performance was linear as Monte Carlo simulations for steady state calculations were embarrassingly parallel^19^ as each particle was transported completely independent of each other.

## 3 RESULTS

### 3.A X‐ray fluence profile at isocenter

**Figure 5:**
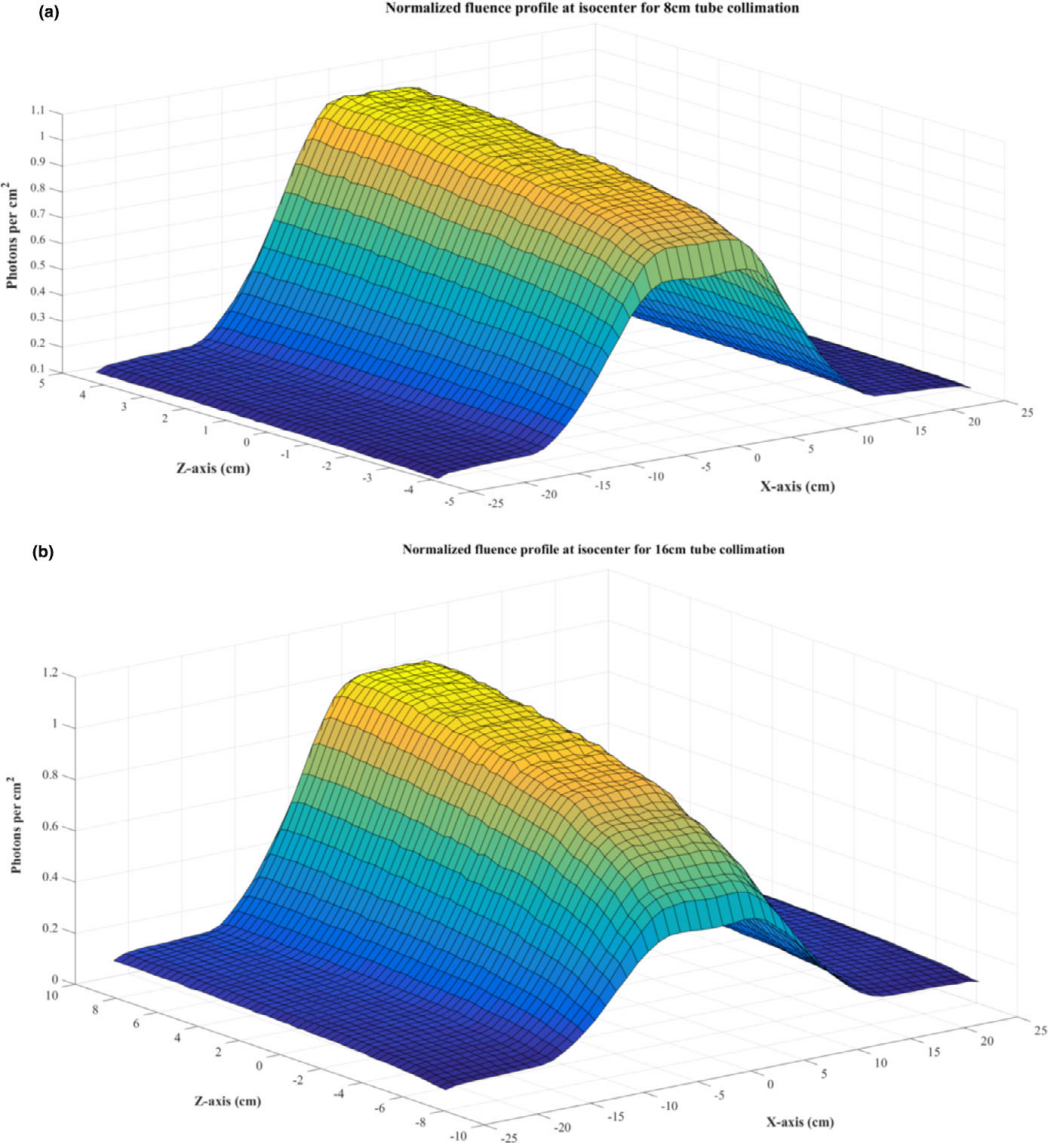
Simulated normalized fluence profile of the x‐ray source at (a) 8 cm and (b) 16 cm tube collimations

Figure 5 shows the normalized profile of the x‐ray fluence tallied at the isocenter of the scanner. The bell shape of the fluence profile along the *x*‐axis is caused by the attenuation due to the bowtie filter. The shape of the fluence profile along the z‐axis is due to the heel effect of the x‐ray tube. The mean standard deviation of the simulated profile from the measurement values was ~0.7% for 8 and 16 cm tube collimations.

### 3.B AAPM Report 195 benchmark tests

Fluka results for the four contiguous cylindrical detectors of case 4 test 1 agreed to better than 1.5% of the benchmark result for both monoenergetic and spectral source at 10 and 80 mm collimations. Fluka results for case 4 test 2 agreed to better than 2% of the benchmark result for the detector in the periphery and better than 1% for the center detector at both 10 and 80 mm collimations. Table 6 shows the relative deviation between Fluka results and the benchmark for all the test problems in case 4.

**Table 6:** Relative deviation of Fluka results in comparison to the benchmark results for Case 4 in the TG 195 report

Test problem	Source	Detector	Rel. deviation
Case 4 test 1	10 mm–56.4 keV	1	0.30%
		2	0.56%
		3	0.77%
		4	0.78%
	10 mm–W/Al 120 kV	1	0.32%
		2	0.63%
		3	1.13%
		4	0.66%
	80 mm–56.4 keV	1	1.21%
		2	1.13%
		3	0.97%
		4	0.63%
	80 mm–W/Al 120 kV	1	1.27%
		2	1.26%
		3	1.07%
		4	0.92%
Case 4 test 2	10 mm–56.4 keV	Center	0.13%
		Perimeter	1.92%
	10 mm–W/Al 120 kV	Center	0.92%
		Perimeter	1.28%
	80 mm–56.4 keV	Center	1.43%
		Perimeter	2.03%
	80 mm–W/Al 120 kV	Center	0.62%
		Perimeter	1.03%

For case 5, the static projection of the monoenergetic source at 0° resulted in a better than 3% agreement between Fluka results and the benchmark for all organs except adrenal gland and breasts for which the results agreed to better than 4%; and for the spectral source, Fluka results agreed to better than 3% for all the organs except the thyroid and adrenal gland which differed by 5% and 6%, respectively. Fluka results for case 5 with tube rotation and monoenergetic source agreed to better than 5% for all the organs except thyroid (9.8%) and breasts (5.4%) in comparison to the benchmark results. For the spectral source, Fluka results agreed to within 5% of the benchmark for 14 of the 17 organs. The results for the adrenal gland, the thyroid and the breasts differed by 8.7%, 7.1%, and 5.9%, respectively. Table 7 shows the relative deviation of Fluka results to the benchmark for all the source types.

**Table 7:** Relative deviation of Fluka results in comparison to the benchmark results for Case 5 test problem in the TG 195 report

Organs	Relative deviation (%)			
	Random rotation		Fixed projection 0°	
	56.4 keV	120 kV	56.4 keV	120 kV
3 soft tissue	1.04%	0.90%	0.99%	0.99%
4 heart	0.80%	0.50%	2.29%	2.37%
5 lung	1.37%	1.09%	1.96%	2.08%
6 liver	4.05%	3.76%	2.32%	2.44%
7 gallbladder	3.93%	4.09%	1.69%	2.60%
8 spleen	3.01%	2.40%	1.48%	2.86%
9 stomach	3.69%	3.49%	1.91%	2.02%
10 large intestine	4.45%	3.17%	1.45%	1.24%
11 pancreas	3.11%	3.02%	1.99%	2.15%
12 adrenal	4.49%	8.66%	4.21%	6.46%
13 thyroid	9.82%	7.12%	2.14%	4.92%
14 thymus	2.92%	3.08%	2.42%	2.35%
15 small intestine	1.93%	2.75%	1.96%	1.51%
16 esophagus	4.22%	4.34%	1.62%	2.55%
17 skin	0.33%	0.38%	0.52%	0.25%
18 breast	5.43%	5.94%	4.22%	2.14%
19 cortical bone	1.06%	0.77%	1.57%	1.71%

Overall, Fluka results agreed to better than 4% to the reference results for the cylindrical phantom simulation and for 14 of the 17 organs in the XCAT phantom simulation.

### 3.C In‐air measurements and simulation

Table 8 shows the simulation results in comparison to the in‐air exposure measurements made using the pencil chamber. The measurement values and the simulation results agreed to within 2% for both 8 and 16 cm tube collimations.

**Table 8:** Air Kerma results measured using the 3 cc computed tomography dose index ion‐chamber at the isocenter of the scanner

Collimation	8 cm	16 cm
Simulation	4.20 cGy	4.98 cGy
Measurement	4.14 cGy	4.89 cGy
Relative error	1.39%	1.79%

### 3.D CTDI phantom measurements and simulation

Table 9 shows the comparison of the simulated exposure values in the CTDI phantom to the measured exposure using the pencil chamber placed at the center hole of the phantom along with the correction for the patient table.

**Table 9:** Simulated and measured Air Kerma at the center of the computed tomography dose index phantom

Collimation	8 cm	16 cm
Simulation without table	0.94 cGy	1.38 cGy
Table correction factor	0.96	0.96
Simulation with table	0.90 cGy	1.32 cGy
Measurement	0.84 cGy	1.23 cGy
Relative error	7.12%	7.64%

The simulation results exceeded the measured exposure by approximately ~7.5% for both tube collimations (7.1% for 8 cm collimation and 7.6% for 16 cm collimation). To correct for this effect, a correction factor (*CF*_*CTDI*_) based on the ratio of the measured and simulated exposure values at the center of the CTDI phantom was calculated:

(6) $$CF^{\mathrm{CTDI}}=\frac{EXP_{\mathrm{measurement}}^{CTDI\;center}}{EXP_{\mathrm{simulation}}^{CTDI\;center}}\;\approx \;0.93.$$

Since the dose in the anthropomorphic phantoms is also measured in a similar scattering environment to a CTDI phantom, the simulation results for the anthropomorphic phantoms were multiplied by the CTDI correction factor (*CF*_*CTDI*_).

### 3.E Physical anthropomorphic phantom measurements and simulation

**Figure 6:**
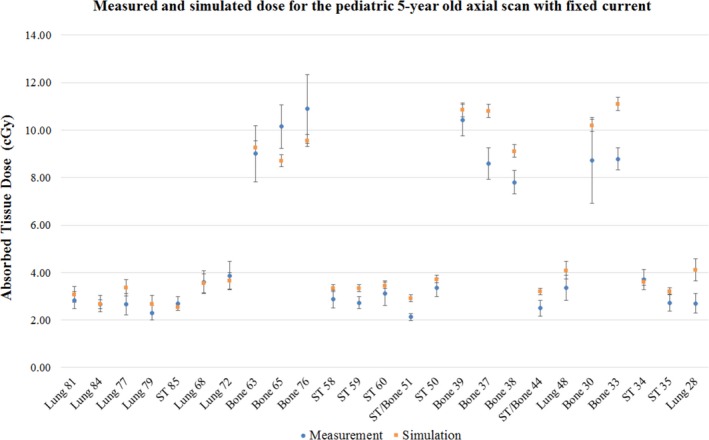
Dose comparison for an axial chest scan of the pediatric phantom at 120 kV and 300 mAs exposure with 95% CI; The abscissa of the plot shows the tissue type and the anthropomorphic phantom hole location number where the MOSFET detectors were loaded in the phantom. The ordinate of the plot shows the absorbed dose in the tissue.

Figure 6 shows the measured and simulated absorbed doses for the pediatric 5‐yr‐old phantom acquired in axial mode. The error bars in the plot represent the 95% confidence interval (CI) of the measurement and simulation doses. The confidence interval of the measurement doses was calculated from the overall measurement error $\left(\sigma_{\mathrm{avg}}\right)$ obtained by propagating the calibration error of the MOSFET detectors in the formula for average measured dose (*D*_*avg*_) from repeated measurements (*D*_1..3_) as shown below:

$$D_{\mathrm{avg}}=\frac{D_{1}+D_{2}+D_{3}}{3};\sigma_{\mathrm{avg}}=\frac{1}{3}\sqrt{{\left(D_{1}*r_{\mathrm{cf}}\right)}^{2}+{\left(D_{2}*r_{\mathrm{cf}}\right)}^{2}+{\left(D_{3}*r_{\mathrm{cf}}\right)}^{2}},$$

Where, *r*_*cf*_ is the relative error in the calibration factor with respect to the measured dose.

The statistical error in the Monte Carlo simulation (*σ*_*MC*_) is used to compute the confidence interval for the simulated doses. The Fluka simulation doses are also corrected with the *CF* ^*CTDI*^ factor. The measured and simulation doses fell within the 95% confidence intervals of each other for 17 of the 25 tissue locations. The results show that except for one lung location (hole 28) and one soft tissue location (hole 59), all soft tissue and lung doses fall within the 95% CI for measurement and simulation. Out of the eight bone locations, only four dose measurements fell within the 95% CI of the simulation results, and two soft‐tissue and bone interface locations (holes 44 and 51) fell outside the 95% CI of the measurement and simulation results.

**Figure 7:**
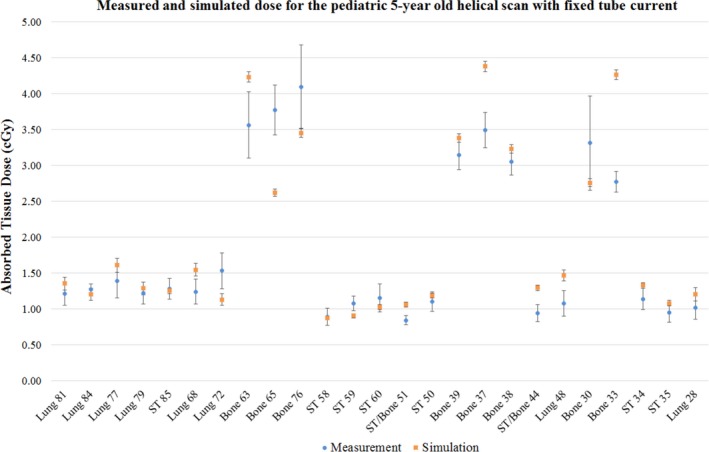
Dose comparison for a helical chest scan of the pediatric phantom at 120 kV and 300 mAs exposure with 95% CI; The abscissa of the plot shows the tissue type and the anthropomorphic phantom hole location number where the MOSFET detectors were loaded in the phantom. The ordinate of the plot shows the absorbed dose in the tissue.

Figure 7 shows the comparison of measurement and simulation doses for a helical scan with fixed tube current. For constructing the 95% CI of the measurement values, the calibration errors of the MOSFETs alone are considered and the deviation due to tube starting position is ignored. Similarly, the 95% CI for the simulation doses are calculated by propagating only the statistical error in the simulation for each of the four tubes start positions (0°, 90°, 180°, and 270°)**.** The results show that except for three lung locations (holes 48, 68, and 73) and one soft tissue location (hole 59), all the soft tissue and lung doses agree within the 95% CI for measurement and simulation. The two soft tissue and bone interface locations (holes 51 and 44) and four bone locations fell outside the 95% CI of the simulation results.

**Figure 8:**
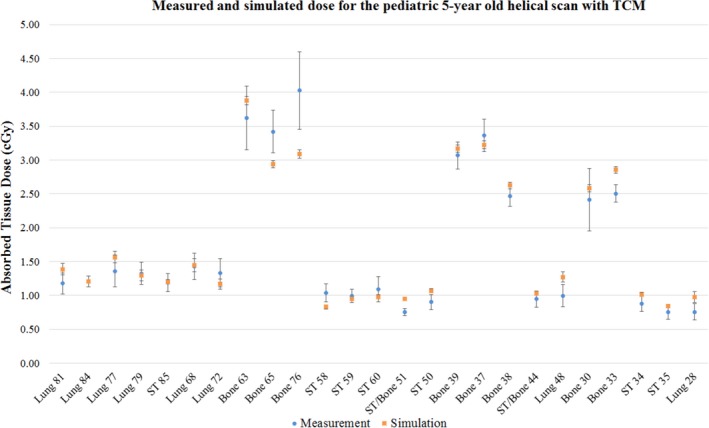
Absorbed dose comparison for helical chest scan of the pediatric phantom with tube current modulation. The abscissa of the plot shows the tissue type and the anthropomorphic phantom hole location number where the MOSFET detectors were loaded in the phantom. The ordinate of the plot shows the absorbed dose in the tissue.

Figure 8 shows the dose comparison for the helical thoracic scan with TCM. The simulated and measured doses fell within the 95% CI of each other for 17 of the 25 MOSFET locations. The doses did not agree for three bone locations (holes 33, 65, and 76), one soft tissue‐bone interface location (hole 51), two soft tissue locations (holes 50 and 58) and two lung locations (holes 28 and 48). When the simulation was performed as a fixed current scan with the average tube current of the TCM scan, only 10 locations agreed within the 95% CI of measured and simulated doses. This indicates that the TCM method implemented here, although simple, is more effective in capturing the dose variations due to the current modulation in the scan. For fixed current and TCM helical scans, when the deviation in the measurements due to tube starting position were taken into account in the uncertainty calculations, the simulation results agreed within the 95% CI of the measurement for all the 25 measurement locations.

**Figure 9:**
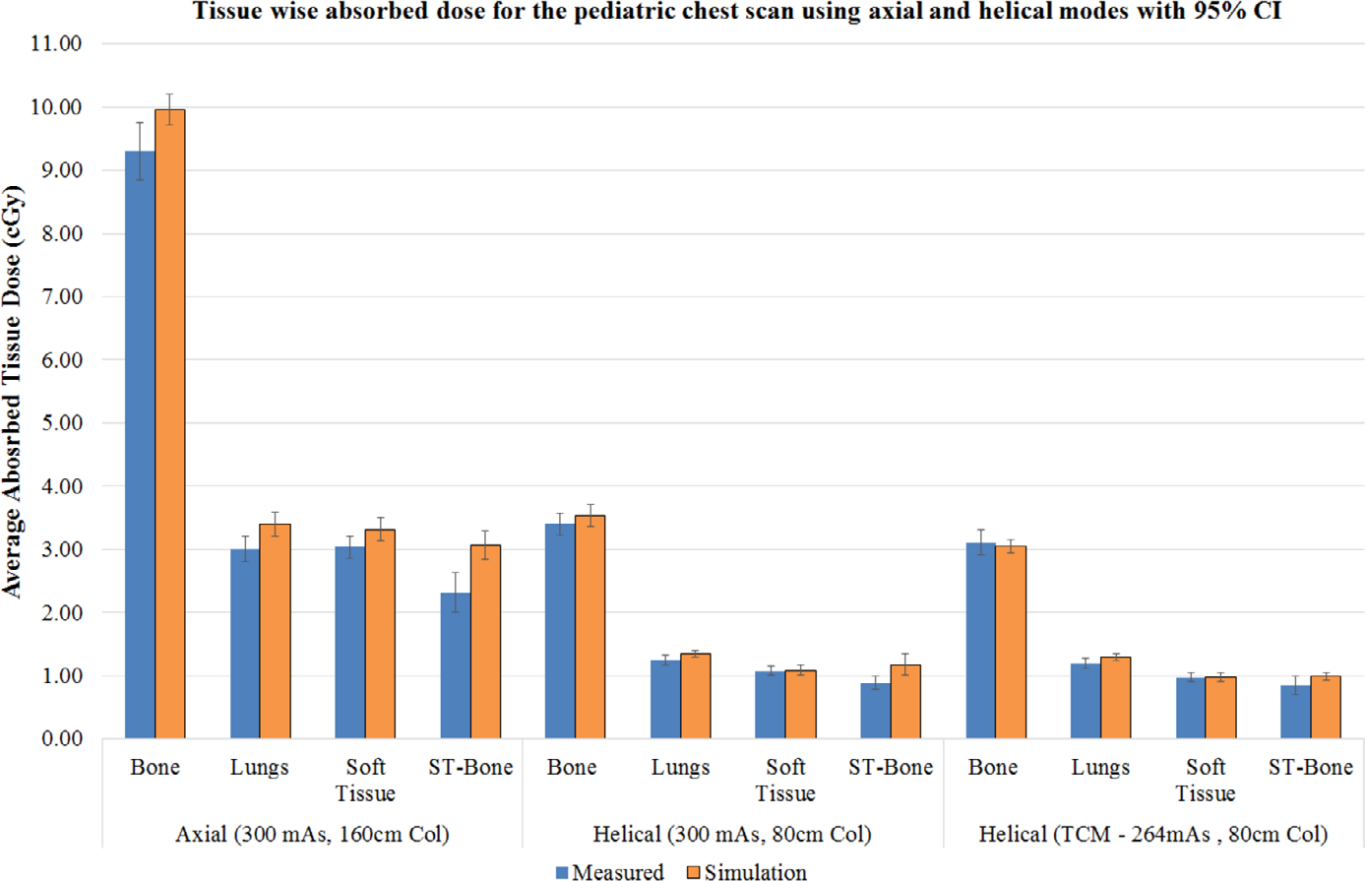
Mean tissue wise absorbed dose comparison for axial, helical, and helical scan with tube current modulation along with 95% CI

Figure 9 shows the tissue wise mean absorbed dose for the pediatric chest scan using the three scan modes. Except for the soft tissue‐bone interface locations, the mean doses for the other tissue types fall within the 95% CI of the measurement and simulation results for both axial and helical scans with fixed current. For the helical TCM scan, all the tissue types including the soft tissue‐bone interface locations fell within the 95% CI of the mean measured and simulated doses.

The computational time for the Fluka simulation on the HPCF for the 5‐yr‐old pediatric phantom was 501, 440, and 684 seconds for the axial, helical, and helical scan with TCM, respectively. Given that the simulations were performed for a particle history of 10^8^ on 100 cores with 10^6^ photons simulated on each core, the mean simulation time across scan types for a particle in a single CPU core was 0.5 ms. The total mean simulation time in the HPCF is ~9 min to achieve statistical errors of <6 % across all the dose tallies. As a comparison, simulation time would be ~45 min if ran using a standalone computer with 20 cores (Xi Computer Corp, San Clemente, CA).

## 4 DISCUSSION AND CONCLUSION

In this study, the characterization of the GE Revolution CT scanner and development of a Fluka Monte Carlo package were created for simulating tissue absorbed doses in both a wide‐beam axial mode (i.e., 160 mm) and 80 mm helical mode with and without TCM. Experimental measurements and computational tools were utilized to quantify the x‐ray spectrum, bowtie filter, beam profile, and primary photon fluence of the scanner using the large body filter and a tube voltage of 120 kV. The validation of the Fluka Monte Carlo package for diagnostic dose calculation in CT examinations was benchmarked using AAPM Report 195 defined tests (4 and 5) and physical measurement test problems. The GE Revolution scanner model was additionally validated using various physical experimental test phantoms. Test problems using a pediatric 5‐yr‐old physical anthropomorphic phantom compared the absorbed dose measured using 25 MOSFET detectors dispersed across the chest region to the simulation results.

Fluka results for the AAPM report 195 case 4 benchmark problem showed that they agree within 2% of the reference results (average of MCNP5, Penelope, Geant4 and EGNsrc results) for all test cases. For the XCAT phantom simulation (AAPM Report 195 case 5), the results agreed to within 4% of the reference results for 14 of the 17 organs across the four different test cases (fixed tube with mono‐energetic and spectral source and rotating tube with mono‐energetic and spectral source). The primary cause for the energy deposition in the adrenal glands to differ by up to 8.7% from the reference results is due to its relatively smaller size resulting in fewer photons depositing energy and a larger statistical error. The breasts and the thyroid, although smaller in size, receive reasonably large energy deposition in comparison to the adrenal glands (an order of magnitude more) and the cause for the larger deviations (up to 5.9% and 9.8%, respectively) from the reference results is likely due to the differences in particle interaction physics between Fluka and other codes. This was further confirmed by a high accuracy simulation (10^9^ photons) of the test problem with spectral source and random tube rotation. When compared to the simulation with 10^8^ photons, the statistical errors for the 10^9^ simulation for the adrenal gland, thyroid, and breasts dropped from 4.8%, 1.2%, and 0.4% to 1.5%, 0.5%, and 0.2%, respectively. The results for the 10^9^ simulation in comparison to the reference means were within 5.5%, 6.5%, and 5.6% for the adrenal gland, thyroid, and breasts. Except for the adrenal gland, the high accuracy simulation did not cause a significant improvement (<1%) to Fluka results in comparison to the reference results. For the adrenal gland there was a 3.1% (5.5% for 10^9^ and 8.6% for 10^8^ histories) improvement.

The discrepancy in the simulated exposure at the CTDI phantom center (leading to the introduction of the *CF* ^*CTDI*^ factor) is due to systematic errors in the estimated x‐ray spectrum. These errors could be due to subtle differences in beam hardening caused by the actual filter materials compared to assumptions made to generate an equivalent spectrum. Air kerma measurements at isocenter agreed well with simulated exposures indicating that the ion chamber does not capture the difference in spectrum when the significant dose contribution is from the primary x‐ray fluence emitted directly from the tube. But for air kerma measurements in the center of the CTDI phantom, a significant dose contribution comes from the secondary radiation caused by scattering interactions in the phantom material. Since the spectral differences are likely to affect the amount of scattered radiation produced, the measurement and simulation results at the center of the phantom could lead to a larger discrepancy.

When comparing the MOSFET measurements in the anthropomorphic phantom to Fluka simulations, locations where the measurement and simulation doses fell outside the 95% CI of each other were at interfaces of soft tissue and bone (e.g., holes 51 and 44, Fig. 6). For the MOSFETS loaded at the interface of soft tissue and bone, the air‐tissue equivalent conversion factor for soft tissue was applied. Additionally, since the MOSFET active region is much smaller than the dose tallies used in the simulation, at the interfaces between tissues, this may have introduced discrepancies between measured and simulated doses. Absorbed doses to the bone in the simulation results are also highly susceptible to the tube starting positions in the helical scans. For the fixed current helical scan, the average deviation in the simulated bone doses for the four simulations with different tube starting positions was 60% while that for lung and soft tissue doses was ~14%. For the measurement doses averaged from three repeat scans with unknown tube starting positions, the deviation in bone was ~23% while that for lung and soft tissue was ~16%. The deviation due to tube starting positions for the simulated bone doses were significantly higher than the uncertainty in the measurement doses. However, since the tube starting positions for the simulations were spaced out uniformly at 90° while the tube starting positions of the measurement scans were unknown, they cannot be compared directly. Also, the main reason for large deviations in the simulated dose at different tube starting positions is due to the small volume of the Monte Carlo tallies that were employed to mimic the MOSFET holes in the phantom. For performing organ dosimetry on real patients, the volume of the Monte Carlo tallies would be equal to the organ volumes and therefore the deviations due to tube starting position will be minimal since more photons will irradiate the target volumes.

To perform patient dose calculations, the patient scans need to be contoured for the radiosensitive organs of interest and once they are contoured, they can be voxelized in Flair and based on the scan settings, patient specific dose calculations can be performed. The model should also be validated for other voltage and filter settings. The TCM model implemented does not take into account the x–y plane modulation in tube current that happens within the patient volume of each reconstructed slice as this information is available in the private DICOM fields that are available currently only to the manufacturer. This is a limitation of the model in its current state and getting access to the x–y plane current modulation would help to improve the dose accuracy of the model for exams using TCM.

This study was designed to incorporate input parameters for one specific CT scanner make and model as a first step towards the ultimate goal of making patient‐specific radiation dosimetry a reality for CT. In an effort to streamline broader implementation of scanner specific Monte Carlo modeling for other CT scanners, the source code from this study will be made available as open‐source code along with all the preprocessing and postprocessing routines necessary to model any CT scanner in Fluka and execute them in high performance clusters. The simulation model developed in this work can be used to benchmark scanners from other manufacturers and to calculate patient organ doses, provided the calibration curve mapping the HU values to tissue composition and scanner specific parameters such as the bowtie profile and beam width are available.

In conclusion, Fluka‐based CT scanner Monte Carlo simulation source model has been developed and the GE Revolution scanner has been characterized using experimental measurements. The model has been validated on benchmark test problems as well as on scanner specific test problems using phantom measurements. The Fluka source model was configured to execute on a high‐performance cluster and the execution times (~9 min) for a pediatric phantom were demonstrated to be feasible for future Monte Carlo based diagnostic dose calculations in a clinical workflow.

## CONFLICT OF INTEREST

The authors declare no conflict of interest.

## References

[1] Brady S , Kaufman R . Investigation of AAPM Report 204 size‐specific dose estimates (SSDE) for pediatric CT implementation. Radiology. 2012b;265:832–840. 23093679 10.1148/radiol.12120131

[2] McCollough CH , Bakalyar DM , Bostani M , et al. Use of water equivalent diameter for calculating patient size and size‐specific dose estimates (SSDE) in CT. Report number 220 of AAPM Task Group 220; 2014. PMC4991550 27546949

[3] Khatonabadi M , Zhang D , Cagnon CH , DeMarco JJ , McNitt‐Gray MF . Water equivalent diameter (Dw) as a patient size metric for estimating organ dose to patients undergoing CT exams. In: RSNA Annual Meeting, (Chicago, IL); 2012.

[4] Moore BM , Brady SL , Mirro AE , Kaufman RA . Size‐specific dose estimate (SSDE) provides a simple method to calculate organ dose for pediatric CT examinations. Med Phys. 2014;41:071917. 24989395 10.1118/1.4884227 PMC5148074

[5] Brady SL , Mirro AE , Moore BM , Kaufman RA . How to appropriately calculate effective dose for CT using either size‐specific dose estimates or dose‐length product. AJR Am J Roentgenol. 2015;204:953–958. 25729893 10.2214/AJR.14.13317

[6] Chen W , Kolditz D , Beister M , Bohle R , Kalender WA . Fast on‐site Monte Carlo tool for dose calculations in CT applications. Med Phys. 2012;39:2985–2996. 22755683 10.1118/1.4711748

[7] Su L , Yang Y , Bednarz B , et al. ARCHERRT ‐ a GPU‐based and photon‐electron coupled Monte Carlo dose computing engine for radiation therapy: software development and application to helical tomotherapy. Med Phys. 2014;41:071709. 24989378 10.1118/1.4884229 PMC4105974

[8] Battistoni G , Bauer J , Boehlen TT , et al. The FLUKA code: an accurate simulation tool for particle therapy. Front Oncol. 2016;6:116. 27242956 10.3389/fonc.2016.00116 PMC4863153

[9] Botta F , Mairani A , Hobbs RF , et al. Use of the FLUKA Monte Carlo code for 3D patient‐specific dosimetry on PET‐CT and SPECT‐CT images. Phys Med Biol. 2013;58:8099–8120. 24200697 10.1088/0031-9155/58/22/8099 PMC4037810

[10] Punnoose J , Xu J , Sisniega A , Zbijewski W , Siewerdsen JH . Technical note: Spektr 3.0—a computational tool for x‐ray spectrum modeling and analysis. Med Phys. 2016;43:4711–4717. 27487888 10.1118/1.4955438 PMC4958109

[11] Turner AC , Zhang D , Kim HJ , et al. A method to generate equivalent energy spectra and filtration models based on measurement for multidetector CT Monte Carlo dosimetry simulations. Med Phys. 2009;36:2154–2164. 19610304 10.1118/1.3117683 PMC2754941

[12] Li X , Samei E , Segars WP , et al. Patient‐specific radiation dose and cancer risk estimation in CT: part I. Development and validation of a Monte Carlo program. Med Phys. 2010;38:397–407. 10.1118/1.3515839 PMC3021562 21361208

[13] Artz NS , Somasundaram E , Brady SL . Limitations of Radiographic FIlm‐Based CT Radiation Beam Profile Measurements. Paper presented at the AAPM 2017 59th Annual Meeting & Exhibition, Denver, CO.

[14] Schneider W , Bortfeld T , Schlegel W , et al. Correlation between CT numbers and tissue parameters needed for Monte Carlo simulations of clinical dose distributions correlation between CT numbers and tissue parameters needed for Monte Carlo simulations of clinical dose distri. Phys Med Biol. 2013;58:4255–4276. 10701515 10.1088/0031-9155/45/2/314

[15] Sechopoulosm I , Ali ES , Badal A , et al. Monte Carlo reference data sets for imaging research: executive summary of the report of AAPM Research Committee Task Group 195. Med Phys. 2015;42:5679–5691. 26429242 10.1118/1.4928676

[16] Berger MJ , Hubbell JH , Seltzer SM , et al. XCOM: Photon Crossections Database.

[17] Brady S , Kaufman R . Establishing a standard calibration methodology for MOSFET detectors in computed tomography dosimetry. Med Phys. 2012a;39:3031–3040. 22755688 10.1118/1.4712221

[18] Chair C‐M , Coffey CW , DeWerd LA , et al. AAPM protocol for 40–300 kV x‐ray beam dosimetry in radiotherapy and radiobiology. Med Phys. 2001;28:868–893. 11439485 10.1118/1.1374247

[19] Delgado MS , Parmeter CF . Embarrassingly easy embarrassingly parallel processing in R: implementation and reproducibility. J Appl Econ. 2013;28:1224–1230.

